# Robot‐Assisted Radical Prostatectomy in a Patient With a Rectal Fistula Following Hydrogel Spacer Placement

**DOI:** 10.1002/iju5.70147

**Published:** 2026-02-02

**Authors:** Erika Ikezoe, Yasukazu Nakanishi, Gaku Okumura, Shu Gozu, Tomonori Kanagawa, Naoki Imasato, Kohei Hirose, Madoka Kataoka, Shugo Yajima, Hitoshi Masuda

**Affiliations:** ^1^ Department of Urology, National Cancer Center Hospital East Chiba Japan

**Keywords:** hydrogel spacer, prostate cancer, rectal fistula, rectal ulcer, SpaceOAR

## Abstract

**Introduction:**

The polyethylene glycol‐based hydrogel spacer (SpaceOAR; Boston Scientific, Marlborough, MA, USA) reduces the rectal dose during prostate radiotherapy and rarely leads to rectal ulceration or fistula formation. We report a case in which robot‐assisted radical prostatectomy (RARP) was successfully performed following this rare complication.

**Case Presentation:**

A 62‐year‐old man developed hematochezia after SpaceOAR placement. Magnetic resonance imaging (MRI) and colonoscopy revealed a rectal ulcer with a fistula. The patient initially received bowel rest and antibiotics, followed by an ileostomy. After endoscopic confirmation of reepithelialization and MRI evidence of hydrogel resorption, the stoma was closed. At his request, the patient subsequently underwent RARP, which was safely performed with transrectal ultrasound guidance.

**Conclusion:**

RARP after a hydrogel‐related rectal fistula is technically feasible; however, it should ideally be performed at an experienced center.

## Keynote Message

- We performed RARP in a patient with prostate cancer complicated by a rectal fistula after hydrogel spacer placement.
- Hydrogel spacers improve the safety of radiotherapy but may rarely lead to severe complications such as rectal fistula.
- Careful follow‐up, timely intervention, and meticulous surgical planning can transform serious complications into manageable events.

**Abbreviations**
CAB: combined androgen blockade
CT: computed tomography
DRE: digital rectal examination
MRI: magnetic resonance imaging
PSA: prostate‐specific antigen
RARP: robot‐assisted radical prostatectomy
RWI: rectal wall infiltration
TRB: transrectal prostate biopsy

## 1 Introduction

Hydrogel spacers are injected between the prostate and rectum prior to radiotherapy to increase the prostate–rectum distance, thereby reducing radiation exposure to the rectum and associated toxicity. Prospective multicenter trials and meta‐analyses have confirmed significant dosimetric benefits with an excellent safety profile [1, 2]. Nevertheless, real‐world data have revealed rare but serious complications. A review of the MAUDE (Manufacturer and User Facility Device Experience) database reported that 35 of 156 adverse events (22.4%) occurred before radiotherapy, including fistulas, sepsis, and embolic events [3]. These findings collectively demonstrate that, although uncommon, severe complications can occur after spacer placement and may interfere with definitive cancer treatment.

Here, we report a case of hydrogel‐related rectal ulceration and fistula in which the patient ultimately underwent robot‐assisted radical prostatectomy (RARP) after stepwise management. This case highlights practical considerations regarding the surgical timing and intraoperative strategies in such settings.

## 2 Case Presentation

A 62‐year‐old man with a history of appendectomy, duodenal ulcers, hypertension, and hyperlipidemia presented with a prostate‐specific antigen (PSA) level of 4.8 ng/mL. Multiparametric magnetic resonance imaging (MRI) revealed a predominantly right‐sided lesion categorized as PI‐RADS 4, with a prostate volume of 20 cm^3^. Transrectal prostate biopsy (TRB) demonstrated adenocarcinoma with a Gleason score of 4 + 4, positive in 6 of 16 cores. Staging computed tomography (CT) and diffusion‐weighted whole‐body imaging revealed no metastases, consistent with clinical stage cT2aN0M0.

**FIGURE 1:**
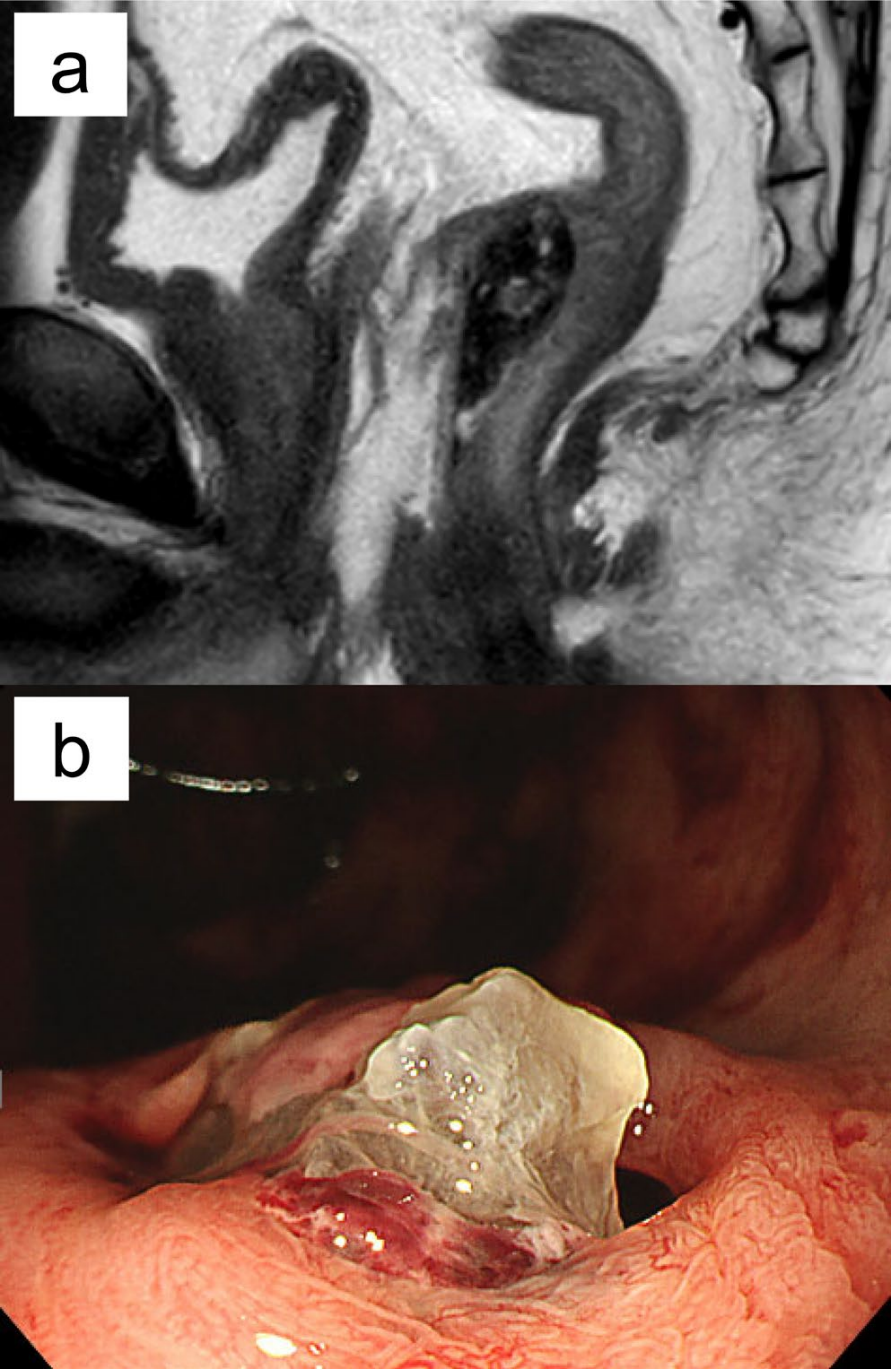
(a) Sagittal T2‐weighted MRI showing hydrogel spacer migration into the anterior rectal wall. (b) Colonoscopy image showing the spacer protrudes from the base of the ulcer, forming a fistula.

The patient was referred to our hospital for radiotherapy, where fiducial gold marker placement and SpaceOAR hydrogel spacer injection were performed. A few days after placement, the patient developed defecation difficulty and a feverish sensation, and 1 month later, hematochezia. Colonoscopy revealed a rectal ulcer, and MRI showed anterior rectal wall infiltration (RWI) by the hydrogel (Figure 1a). Conservative management—including bowel rest and antibiotics—was initiated; however, the patient developed progressive hydrogel protrusion, recurrent bleeding, and suspected fistula formation (Figure 1b). The radiotherapy schedule was canceled. A laparoscopic ileostomy was performed to minimize intra‐abdominal adhesions and reduce the risk of anastomotic leakage. Combined androgen blockade (CAB) had already been initiated at the previous hospital 3 months before the planned radiotherapy and was continued during recovery.

**FIGURE 2:**
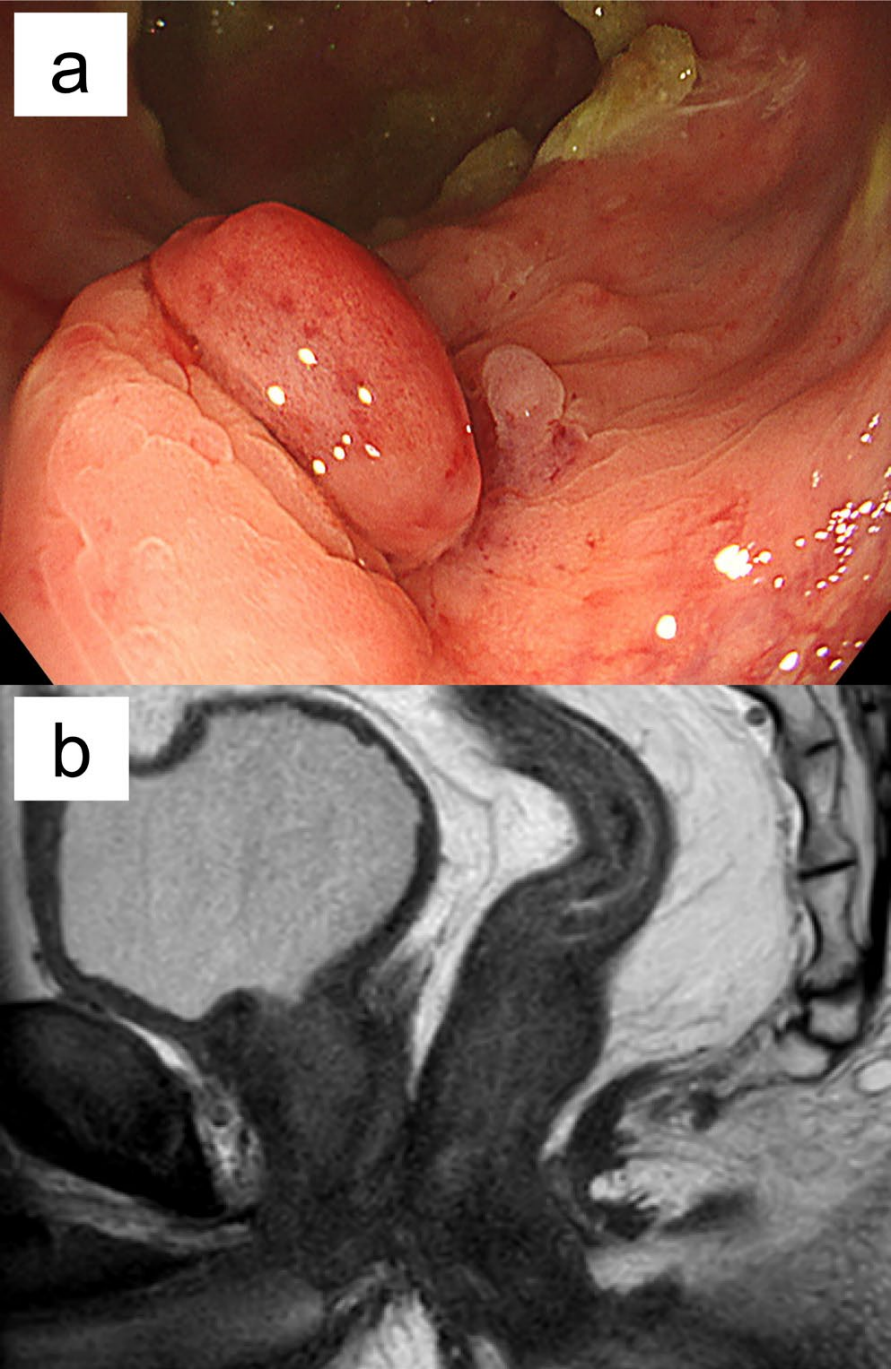
(a) Colonoscopy showing granulation tissue formation. No surrounding fistula or mucosal defect; scarring is present. (b) Sagittal T2‐weighted MRI showing resolution of previously observed low signal in the anterior wall.

Follow‐up colonoscopies and MRI were performed at regular intervals. 8 months after ileostomy, colonoscopy confirmed complete epithelialization and scarring of the ulcer, without residual fistula or mucosal defects (Figure 2a). After 10 months, MRI demonstrated complete hydrogel resorption (Figure 2b). Subsequently, ileostomy closure was performed.

**FIGURE 3:**
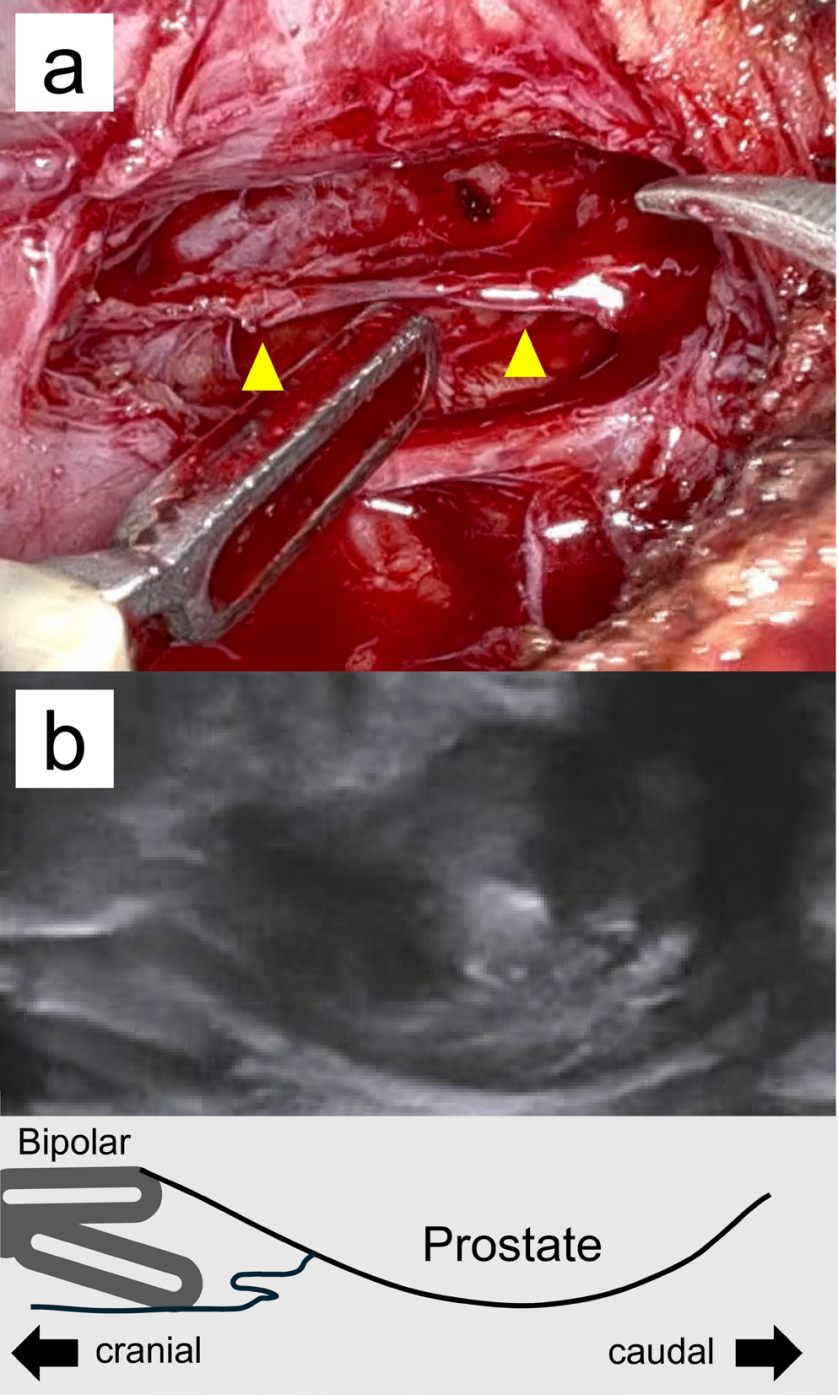
(a) The Denonvilliers' fascia detachment layer in this case (yellow arrow). (b) Sagittal transrectal ultrasound image (top) and its simplified schematic (bottom). These illustrate prostate dissection guided by transrectal ultrasonography and digital rectal examination. The posterior plane was reestablished closer to the prostatic capsule than in standard procedures.

After the rectal ulcer had resolved, treatment options were reconsidered and discussed again with the patient. Given the history of rectal ulceration and the patient's reluctance to undergo radiotherapy directed at the previously ulcerated rectal area, together with the fact that SpaceOAR interposition was no longer feasible, the patient was concerned about the increased risk of hemorrhagic radiation proctitis and therefore expressed a preference to avoid radiotherapy. As a result, the patient opted for RARP. Using the da Vinci Xi Surgical System (Intuitive Surgical Inc., Sunnyvale, CA, USA) with a six‐port transperitoneal approach, dissection between the prostate and rectum was carried out along the prostatic side. Dissection proceeded in the plane of Denonvilliers' fascia covering the anterior rectal wall (Figure 3a). To enhance safety, this step was guided by transrectal ultrasonography and digital rectal examination (Figure 3b). No adhesions were observed at the base; however, dense apical adhesions required sharp dissection (Video S1).

The total operative time was 138 min, console time was 104 min, and estimated blood loss was 28 mL. Cystourethrography on postoperative day 7 showed no urinary leakage at the vesicourethral anastomosis, and the catheter was removed as planned. The patient was discharged on postoperative day nine without any complications. Pathological examination revealed no residual carcinoma. The PSA was undetectable preoperatively and remained so at 3 months postoperatively. Urinary continence improved to pad‐free at 3 months.

## 3 Discussion

SpaceOARs are widely used to reduce rectal radiation exposure during prostate radiotherapy. Although clinical trials have confirmed an excellent safety profile, rare but serious complications—including rectal ulceration and rectourethral fistula—have been reported and are often linked to misinjection or RWI [3, 4]. An analysis of the MAUDE database identified six cases of rectal injury occurring prior to radiotherapy that required surgical intervention [3].

RWI is a sentinel event that precedes many complications. Risk is independently increased by two factors: (1) a history of TRB, which carries a higher risk than transperineal biopsy, and (2) a small prostate volume, which limits the available perirectal space [5, 6]. In this case, both risk factors and operator inexperience likely contributed, emphasizing the need for careful post‐insertion imaging.

Both radical prostatectomy and radiotherapy were treatment options. After rectal fistula formation, tissue fragility from fibrosis and ischemia, as well as the risk of fistula recurrence, must be considered for either approach. Although there has been a report in which radiotherapy was selected, no mention has been made of long‐term outcomes [7]. Reports of RARP after spacer‐related complications are scarce. Kuperus et al. [8] first described successful fistula repair with concomitant RARP, omental interposition, and diversion. Subsequent reports documented the feasibility of RARP after spacer placement, highlighting modifications in posterior dissection [9, 10].

In our case, surgery was performed only after complete mucosal reepithelialization, full hydrogel resorption, and fibrosis maturation were confirmed. Posterior dissection was performed intrafascially above Denonvilliers' fascia and close to the prostate capsule, using sharp, energy‐limited techniques to minimize traction on fragile scar tissue. These maneuvers were consistent with previous recommendations [9, 10], and were enhanced by real‐time imaging guidance. Intraoperative navigation deserves emphasis: both transrectal ultrasonography and serial digital rectal examination proved valuable when the posterior plane was distorted [11, 12]. Minimal blood loss and the absence of rectal injury in our case support the feasibility of a carefully tailored RARP approach once adequate healing is established.

The optimal timing of surgery remains debated. Early RARP may take advantage of the lubricating effect of the undegraded gel but risks operating in an inflamed field, potentially compromising fistula repair. In contrast, delayed RARP allows for complete ulcer healing and hydrogel absorption but involves navigating dense fibrosis. The favorable oncological and functional outcomes in our patient suggest that delayed surgery is a reasonable option when rectal integrity is confirmed preoperatively via imaging and endoscopy.

Adhesion severity varies based on hydrogel infiltration and the inflammatory response, underscoring the need for individualized surgical planning. Further case accumulation is necessary to evaluate reproducibility. Such complex procedures should be performed at experienced centers by surgeons proficient in robotic techniques.

As this was a single case, the generalizability of delayed RARP following hydrogel‐related rectal injury remains uncertain.

## 4 Conclusion

By confirming complete rectal healing and full hydrogel absorption before surgery, RARP was safely performed following a spacer‐related rectal fistula. Minimal blood loss, no residual carcinoma, and favorable continence recovery support delayed RARP as a viable treatment option for carefully selected patients at experienced institutions.

## Consent

The authors have nothing to report.

## Conflicts of Interest

The authors declare no conflicts of interest.

## Supporting information


**Video S1:** Severe adhesion observed at the apex of the prostate.

## Data Availability

The data that support the findings of this study are available on request from the corresponding author. The data are not publicly available due to privacy or ethical restrictions.
